# Cephalosporins-induced intestinal dysbiosis exacerbated pulmonary endothelial barrier disruption in streptococcus pneumoniae-infected mice

**DOI:** 10.3389/fcimb.2022.997368

**Published:** 2022-08-24

**Authors:** Jia-Feng Wang, Chang-Yi Shi, Hua-Zhong Ying

**Affiliations:** Key Laboratory of Experimental Animal and Safety Evaluation, Hangzhou Medical College, Hangzhou, China

**Keywords:** community-acquired infection, endothelial barrier, endotoxin, probiotic, butyrate

## Abstract

Antibiotic abuse is growing more severe in clinic, and even short-term antibiotic treatment can cause long-term gut dysbiosis, which may promote the development and aggravation of diseases. Cephalosporins as the broad-spectrum antibiotics are widely used for prevention and treatment of community-acquired respiratory tract infection in children. However, their potential consequences in health and disease have not been fully elaborated. In this study, the effects of cefaclor, cefdinir and cefixime on intestinal microbiota and lung injury were investigated in *Streptococcus pneumoniae* (Spn)-infected mice. The results showed that the proportion of coccus and bacillus in intestinal microbiota were changed after oral administration with cefaclor, cefdinir and cefixime twice for 10 days, respectively. Compared with the Spn-infected group, the proportion of *Bifidobacterium* and *Lactobacillus* in intestine were significantly reduced, while *Enterococcus* and *Candida* was increased after cephalosporin treatment. Furthermore, 3 cephalosporins could obviously increase the number of total cells, neutrophils and lymphocytes in BALF as well as the serum levels of endotoxin, IL-2, IL-1β, IL-6 and TNF-α. Mechanically, cephalosporins accelerated Spn-induced pulmonary barrier dysfunction *via* mediating the mRNA expressions of endothelial barrier-related proteins (Claudin 5, Occludin, and ZO-1) and inflammation-related proteins (TLR4, p38 and NF-κB). However, all of those consequences could be partly reversed by *Bifidobacterium bifidum* treatment, which was closely related to the elevated acetate production, indicating the protective effects of probiotic against antibiotic-induced intestinal dysbiosis. Therefore, the present study demonstrated that oral administration with cephalosporins not only disrupted intestinal microecological homeostasis, but also increased the risk of Spn infection, resulting in severer respiratory inflammation and higher bacterial loads in mice.

## Introduction

Streptococcus pneumoniae (Spn) is an important pathogen of community-acquired infections and a common cause of respiratory infections with high rates of death, which can cause otitis media, sinusitis, meningitis, bacteremia and sepsis in children, the elderly and immunodeficient patients (Engholm et al., 2017; Lanks et al., 2019). According to WHO data, approximately 1.6 million people die from Spn infection worldwide each year, including 1 million children under 5 years of age, and more than 90% of deaths occurring in developing countries (Mathur et al., 2018). Oral cephalosporins are commonly used in pediatric clinics to treat Spn infections, but emerging evidence display that the intervention with antibiotics during Spn infection can interfere with the colonization of the intestinal microbiota (Diallo et al., 2020; Larsson et al., 2021; von Specht et al., 2021). Even short-term antibiotic treatment can cause long-term gut dysbiotic states, which may cause endothelial barrier disruption of both intestinal and lung tissues (Trivedi and Barve, 2020; Andremont et al., 2021).

Gut microbiota is composed of various microorganisms, such as bacteria, viruses and fungi in the intestine, and their living environment, which affect the physiological functions and pathological changes of host cells (Chen et al., 2021). The intestinal microbiota has been taken attention increasingly because of its crucial roles in human immunity, metabolism, endocrine, neurodegeneration and other physiological processes (Michaudel and Sokol, 2020; Jia et al., 2021; Murciano-Brea et al., 2021; Xu et al., 2020). Patients with respiratory diseases, including allergic asthma, acute lung injury, pulmonary fibrosis, and bacterial infection, often have drastic changes of the proportion and function of intestinal and lung microbiota (Zhou et al., 2021). Intestinal dysbiosis affects respiratory immunity and barrier function through the microbiota-gut-lung axis, resulting in the reduction of the pulmonary resistance to Spn; in turn, pulmonary barrier disruption aggravates the development of acute lung injury and causes intestinal dysfunction through excessive production of cytokine storms (Schuijt et al., 2016; Wang et al., 2018). Under normal conditions, the intestinal microbiota is relatively constant, but it is susceptible to be changed by various environmental factors, especially the antibiotic abuse (Ianiro et al., 2016). Oral cephalosporins as the most widely used antibiotics for treating Spn infection will inevitably lead to the dysbiosis in gut-lung axis and alter host immune status (Becker et al., 2016). However, whether the aggravation of bacterial pneumonia is the primary cause or precipitating cause induced by increased pathogen acquisition have not been well clarified. Therefore, in this study, the intestinal microbiota was depleted by using 3 different cephalosporins cefaclor, cefdinir and cefixime, respectively, and then the mice were infected intranasally with Spn to investigate the host inflammatory response and pulmonary hyperpermeability.

## Materials and methods

### Reagents

Cefaclor tablets, cefdinir powers and cefixime tablets were purchased from Yabang Pharmaceutical Co. Ltd., Shanghai Aladdin Biochemical Technology Co. Ltd., and Tianjin Pharmaceutical Co. Ltd., respectively. *Bifidobacterium bifidum* (FXJCJ9M10) was purchased from Xi’an Victory Biochemical Technology Co. Ltd., China.

Spn (ATCC 49619) was obtained from Peking BeNa Biotech Co. Ltd., China. It was inoculated in Muller-Hinton agar (MH) culture mediums at 37°C for days until the bacteria presented logarithmic growth. The MH mediums was centrifuged (12,000 r/min for 2 min) and then the bacterial suspension was diluted by sterile normal saline and the final concentration of the bacterial suspension was adjusted to 1.5×10^9^ by using a turbidimeter (Dichen Biotech Co. Ltd., China).

### Spn-infected model and antibiotic treatment

The male ICR mice (body weight 16-18 g) were obtained from Zhejiang Laboratory Animal Center. The experiment project was approbated by the ethics committee of Hangzhou Medical College, and the approval number was 2021R10-003. The mice bred in a close system and individually ventilated cages for 1 week to adapt themselves to the new situations.

**Figure 1:**
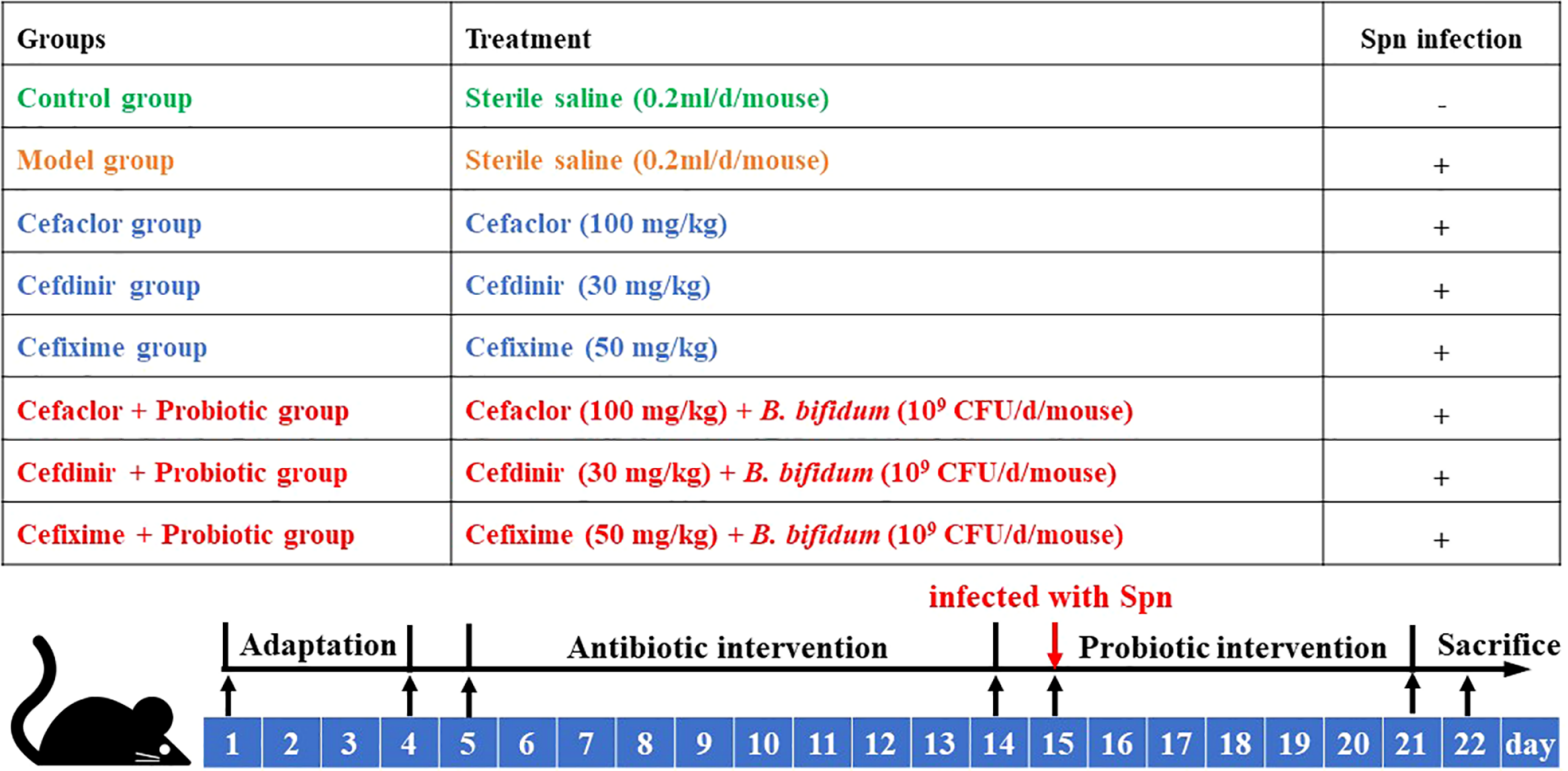
The flow of animal experiment.

The mice were randomly divided into 8 groups and the method of Spn-infected modeling was described as shown in **Figure 1**. The doses of cephalosporins used in the study were established according to their clinical doses in the manufacturer’s instructions. Each group had 20 mice. Except the mice in the control group and model group, others were pre-administrated orally with corresponding cephalosporins twice for 10 days. After anaesthetized by inhalation of ether in operation, except the mice in the control group, other mice were challenged intranasally with 50 μl of Spn suspension (6 × 10^8^ CFU/ml) as previous studies reported (Kim et al., 2019; Hu et al., 2021). And the mice in cephalosporin and *B. bifidum*-co-treated groups were administrated alone with *B. bifidum* suspension (2.5 × 10^8^ CFU) for 7 days (Lu et al., 2021). Twelve hours after the last probiotic administration, ten mice were anesthetized by isoflurane and killed. The lung tissues and blood were collected for the detection of related indicators.

### Observation on the pathological changes of lung tissues in mice

The upper lobe of the right lung tissues of mice in each group was fixed with 10% formaldehyde for 24 hours, soaked in 75% ethanol for 24 hours, embedded in paraffin, and sectioned at 4 μm. The lung tissue was stained with htoxylin-eosin dye and the pathological changes were observed under the microscopes and imaging systems (Leica, Germany). The pathological changes were assessed by using the grading system of 1 (degree, 0), 2 (degree, 1%-25%), 3 (degree, 26%-50%), 4 (degree, 51%-75%) and 5 (degree, 76%-100%) as previous study (Takenaka et al., 2006).

In addition, the expression of TLR4 in lung tissues was detected by the immunohistochemical method, and the processed were operated according to the instructions of the kits. Three high-power visual fields were randomly selected from each slice and calculated by the microscopes and imaging systems (Leica, Germany), and the mean value was taken for further analysis.

### Determination of pulmonary hyperpermeability

Two hours after the last probiotic administration, 5 mice in each group were randomly selected, weighed and injected with 2% Evan Blue (20 mg/kg body weight) in the tail vein. About 30 min after anesthetized by isoflurane, the mice were killed, the whole lung tissues were obtained and washed with PBS to remove blood around the lungs. Then, 100 mg of lung tissues was taken and mixed with 3 ml of acetone-saline solution (volume 7:3) in a 37°C incubator for 24 hours. Three milliliters of supernatant were added into the cuvette and the absorbance values of the samples at 620 nm were detected by using an ultraviolet spectrophotometer (PerkinElmer, USA) to reflect the pulmonary hyperpermeability as previously reported (Yu et al., 2020).

### Cell count in bronchoalveolar lavage fluid

At the day 3^rd^ after Spn infection, five mice in each group were executed. About 0.3 ml of sterile PBS was aspirated from the trachea into the lungs with a syringe each time, and BALF was collected by gently drawing back and repeated three times. The collected BALF was centrifuged at 4°C, 3000 r/min for 10 min, and the upper layer was transferred to a clean plastic pipe and stored at -20°C. The lower layer of cell precipitate was added with erythrocyte lysate, centrifuged at 4°C, 3000 r/min for 10 min, and resuspended with 200 μl of PBS for cell count. Under the light microscope, the number of cells in different colors was counted, and the changes in the ratio of macrophages, neutrophils and lymphocytes were calculated.

### Determination of pro-inflammatory cytokines in serum

Two hours after the last probiotic administration, ten mice were anesthetized by isoflurane. The blood was obtained from abdominal aorta and kept in tubes at 4°C for 4 hours. After the clotted blood was centrifuged, 3000 r/min for 10 min, the supernatant was collected. The levels of pro-inflammatory cytokines (IL-2, IL-1β, IL-6 and TNF-α) and endotoxin in serum were detected by using commercialized ELISA kits (Lioke Biotech Co. Ltd., China) according to the manufacturer’s instructions.

### Real-time quantitative PCR assay

Total RNA was extracted from right lung tissue by using RNA extraction kit (Beyotime Biotech Co. Ltd., Shanghai, China), and its concentration and purity were measured by UV spectrophotometer (PerkinElmer, USA). cDNA was obtained by reverse transcription and stored at -20°C. The fragments of ZO-1, Claudin 5, Occludin, TLR4, p38 and NF-κB mRNA were amplified by real-time quantitative PCR (RT-qPCR). The sequences of the primers used in the study were shown in **Supplementary Table S1**. The reaction system was employed as follows: 10.0 μl, ROX Reference Dye II (50×); 0.4 μl, cDNA (50 ng/μl); 2.0 μl, upstream primers (10 μmol/L); 0.8 μl, downstream primers (10 μmol/L); 6.0 μl, ddH_2_O. The specific operation was carried out strictly according to the kit instructions. Reaction conditions was employed as follows: 95°C for 30 s; 95°C for 5 s, 60°C for 34 s, 40 cycles. The relative expression levels were quantified by the 2^-ΔΔCt^ method.

### Determination of bacterial loads in lung tissues

About 0.1 g of lung tissues was added into 9 ml of sterile saline and ground well by using a homogenizer. One milliliter of homogenate was taken and then mixed with 9 ml of sterile saline as the test sample. Subsequently, 1 ml of the diluted homogenate was added into a sterile flat dish which contained sterile MH mediums with 5% defibrinated sheep blood. Each sample was measured three times. The dishes were kept in an incubator (Thermo, USA) at 37°C with 5% CO_2_ and the number of colonies was counted after 24 h of incubation.

### Determination of intestinal microbiota

After anesthetized by isoflurane, the contents of the intestine were taken as 0.5 g under aseptic conditions. The contents were diluted 10 times with saline as the test solution. Each dilution (100 μl) was daubed with a glass applicator on the surface of different culture mediums, including blood agar plates, MacConkey plates, Enterococcus plates and Candida color plates. Blood agar plates are used to culture Campylobacter and Helicobacter pylori for 24 h; MacConkey plates culture Gram-negative enterobacteria for 24 h; Enterococcus plates culture Enterococcus for 24 h; and Candida plates culture fungi for 48 h. Three plates were made for each concentration of each specimen and incubated in a CO_2_ incubator at 37°C. The number of colonies on each plate was counted and the average number of colonies on each gradient was calculated. Bacterial quantification was performed according to the following formula: tissue bacterial content (CFU/g) = number of colonies × dilution times/tissue weight. Each type of bacteria is initially identified according to Gram staining and colony morphology, and then identified by a bacterial auto-analyzer (Hangzhou ZEXI Biotech Co. Ltd., China).

### The levels of short chain fatty acids in intestinal contents

The levels of SCFAs in intestinal contents were detected by GC-MS assay as previous study (Chen et al., 2019). The appropriate amounts of acetic acid, propionic acid and butyric acid were weighed and mixed with ultrapure water in a 10 ml flask as the standard solution. The standard curves were calculated and plotted according to the chromatograms of the corresponding concentrations. The mouse feces (1.0 g) were weighed and added to 2 ml of ultrapure water. The mixture was mixed thoroughly and centrifuged for 10 min at 1 000 r/min. The supernatant was passed through a 0.22 μm filter membrane for GC-MS analysis, and the contents of ethylene, propionic and butyric acids in mouse feces were calculated.

The levels of SCFAs were detected on an Agilent DB-FFAP125-3232 column (30 m × 250 μm × 0.25 μm) by using the Thermo Trace1310 GC-MS system (Thermo, USA) as follows: carrier gas was He. Flow rate was 1 mL/min. The column temperature was first set at 80°C for 1 min and then speeded at 10°C/min to 200°C. The detector temperature was controlled at 230°C, and the inlet temperature was 200°C. The flow rates of hydrogen, air and nitrogen were set at 30, 300 and 20 ml/min, respectively. The injection volume was 1 μl and the analysis time was 13.5 min for each sample.

### Statistical analysis

Statistical analysis was performed by using GraphPad Prism 8.0 software. Data were expressed as mean ± standard deviation (SD). One-way ANOVA and Turkey tests were used for comparison between groups. P<0.05 indicated that there was statistically significant difference.

## Results

### Cephalosporins exacerbated Spn-induced acute lung injury in mice

**Figure 2:**
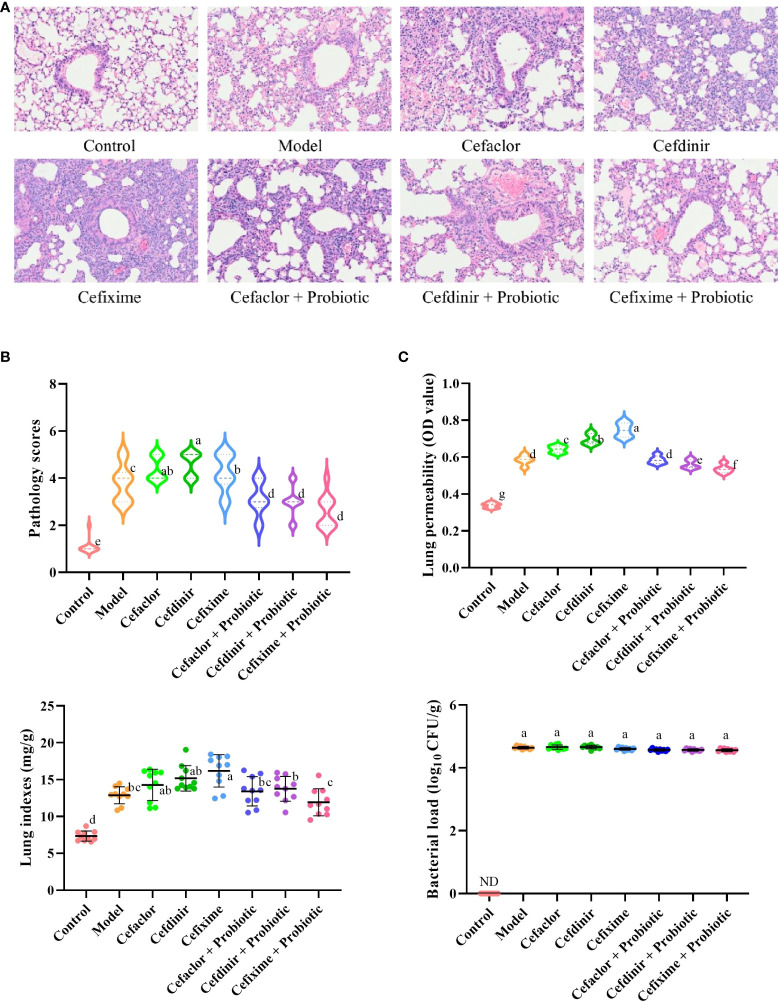
Effects of cephalosporins on Spn-induced acute lung injury in mice. **(A)** The histopathological features of the lung tissues in Spn-infected mice. **(B)** The levels of lung injury were estimated by pathological scores. **(C)** Effects of cephalosporins on pulmonary hyperpermeability, lung indexes and bacterial loads in infected mice. ND, not detected. Data were showed as mean ± SD. Different letters indicated statistically significant differences, P <0.05.

Previous reports had demonstrated that there was no susceptibility difference to S. pneumonia upon the C3H/HeN, C57BL/6, and ICR mice (Takashima et al., 1996; Kim et al., 2019). Thus, ICR mice were used in this study for investigation of Spn infection. As shown in **Figure 2**, the alveolar structure of lung tissues in the control group was observed to be intact under the microscope, while the model group showed marked exudation of inflammatory cells from the alveolar lumen, interstitial edema, ruptured capillary walls and thickened alveolar walls. However, inflammatory responses in the lung tissues of the cephalosporins-treated groups were more serious, and also the pathology scores, lung permeabilities and lung indexes were dramatically increased (*P <*0.05) compared with those in model group. In the probiotic-treated groups, the alveolar structure was relatively intact, the alveolar lumen exudate was significantly reduced, the degree of interstitial oedema was decreased and the permeability was lightened (*P <*0.05), indicating that probiotic could alleviate the acute lung damage caused by Spn infection.

In addition, the bacterial loads in the model group were sharply elevated after Spn infection, while it could not be detected in the control group. However, neither cephalosporin pre-treatment nor probiotic intervention could reduce the bacterial loads in the lung tissue of Spn-infected mice (*P >*0.05). It indicated that long-term pre-treatment with cephalosporin could induce intrinsic drug resistance and probiotic intervention could not inhibit the growth of Spn in the lung tissue of infected mice.

### Cephalosporins exacerbated Spn-induced inflammatory responses in mice

**Figure 3:**
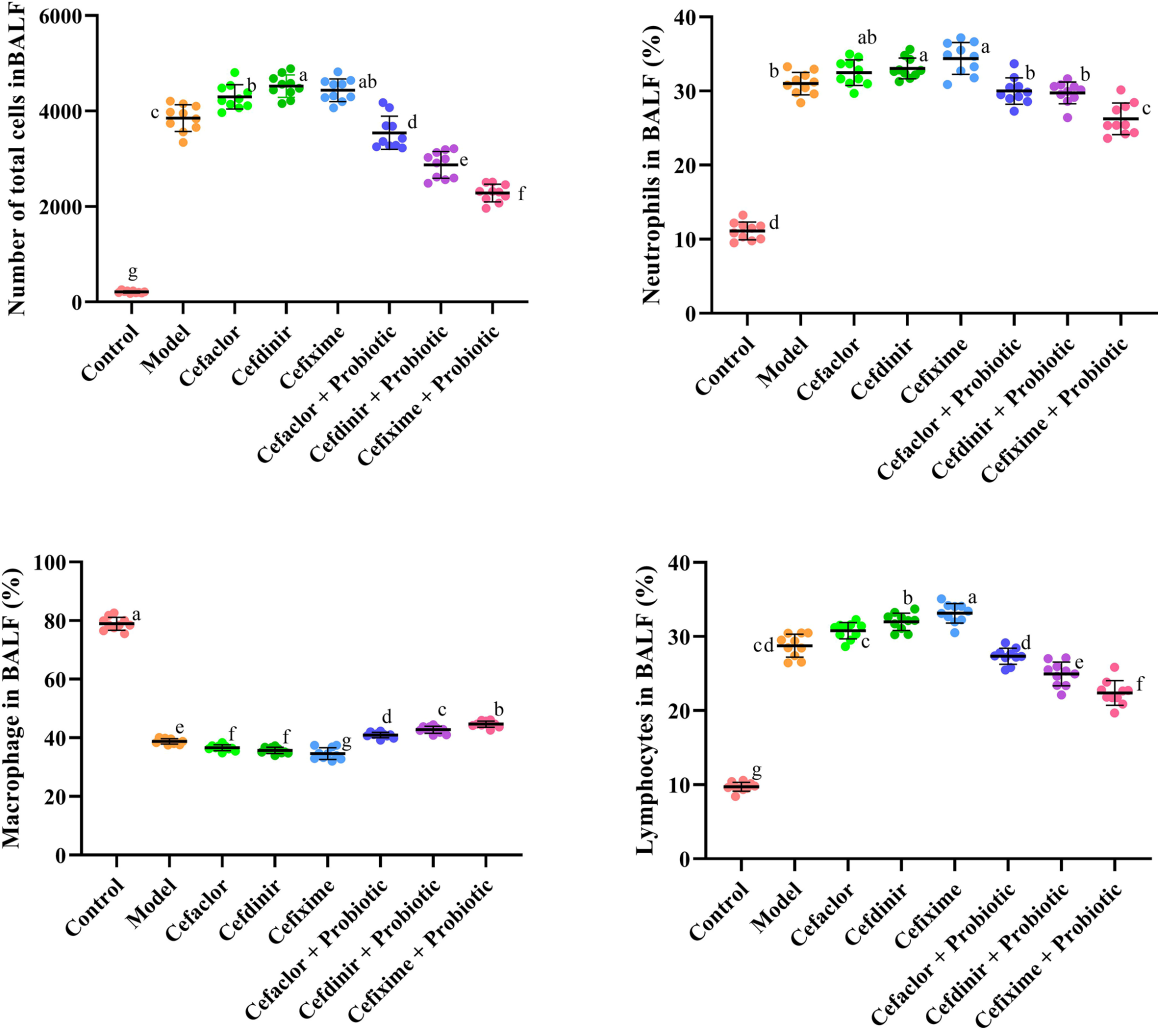
Effects of cephalosporins on the recruitment of inflammatory cells in the BALF of Spn-infected mice. Data were showed as mean ± SD. Different letters indicated statistically significant differences, P <0.05.

**Figure 4:**
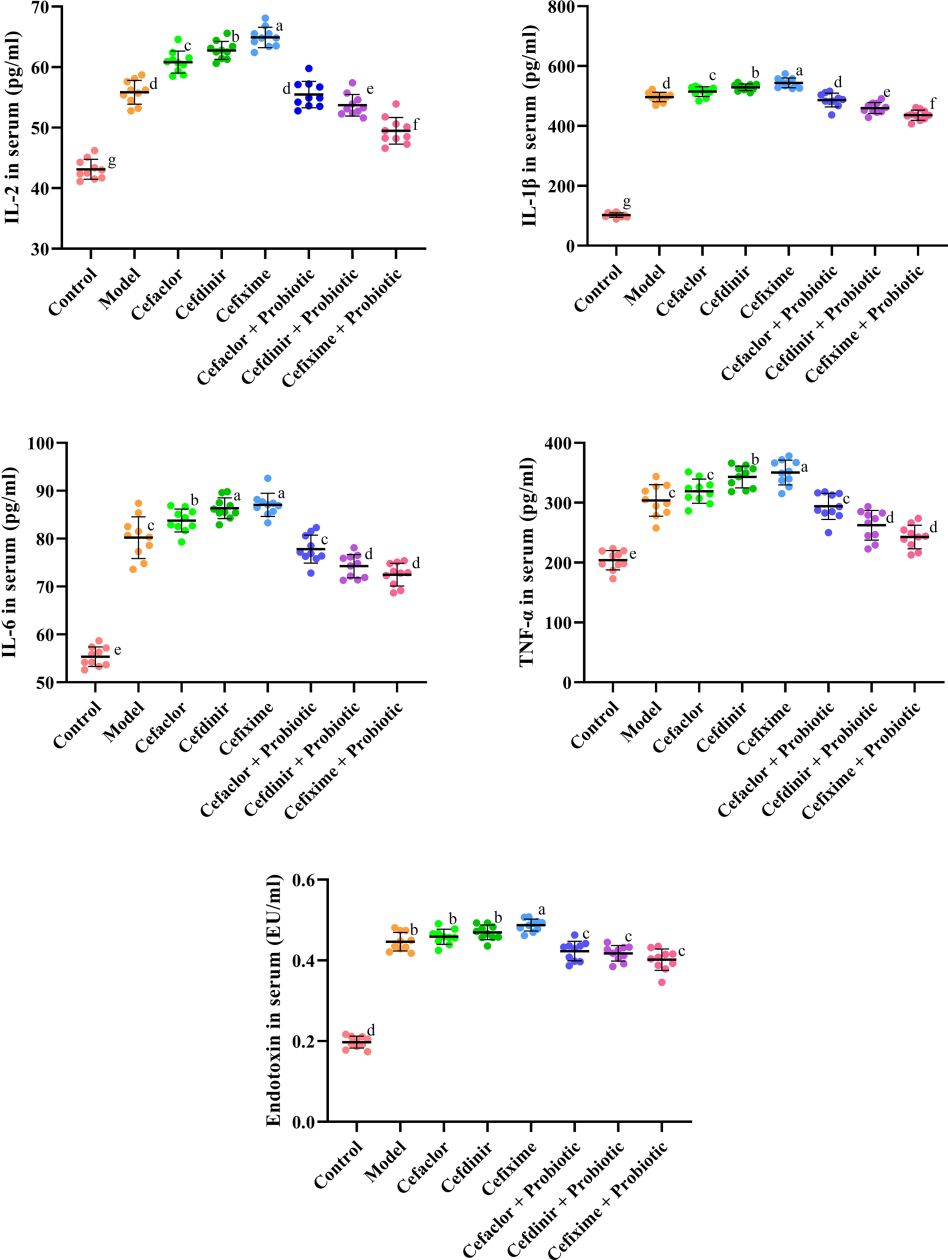
Effects of cephalosporins on the production of pro-inflammatory cytokines and endotoxin in the serum of Spn-infected mice. Data were showed as mean ± SD. Different letters indicated statistically significant differences, P <0.05.

As shown in **Figures 3** and **4**, the number of total cells, Neutrophils, macrophages, and lymphocytes in BALF as well as the levels of pro-inflammatory cytokines (IL-2, IL-1β, IL-6 and TNF-α) and endotoxin in serum were significantly increased in the model group (*P <*0.05), compared with these in the control group. However, the levels of those indexes in cephalosporins-treated groups were much higher than those in the model group (*P <*0.05), indicating that long-term antibiotic intervention could induce gut dysbiosis and thereby aggravate Spn-induced inflammatory responses in mice. However, treatment with probiotic could variably decline the levels of IL-2, IL-1β, IL-6, TNF-α and endotoxin in serum. It also inhibited the exudation of total cells, Neutrophils, macrophages, and lymphocytes in BALF. These results indicated that probiotic intervention weakened antibiotic-exacerbated inflammatory responses induced by Spn.

### Cephalosporins exacerbated intestinal dysbiosis in mice

**Figure 5:**
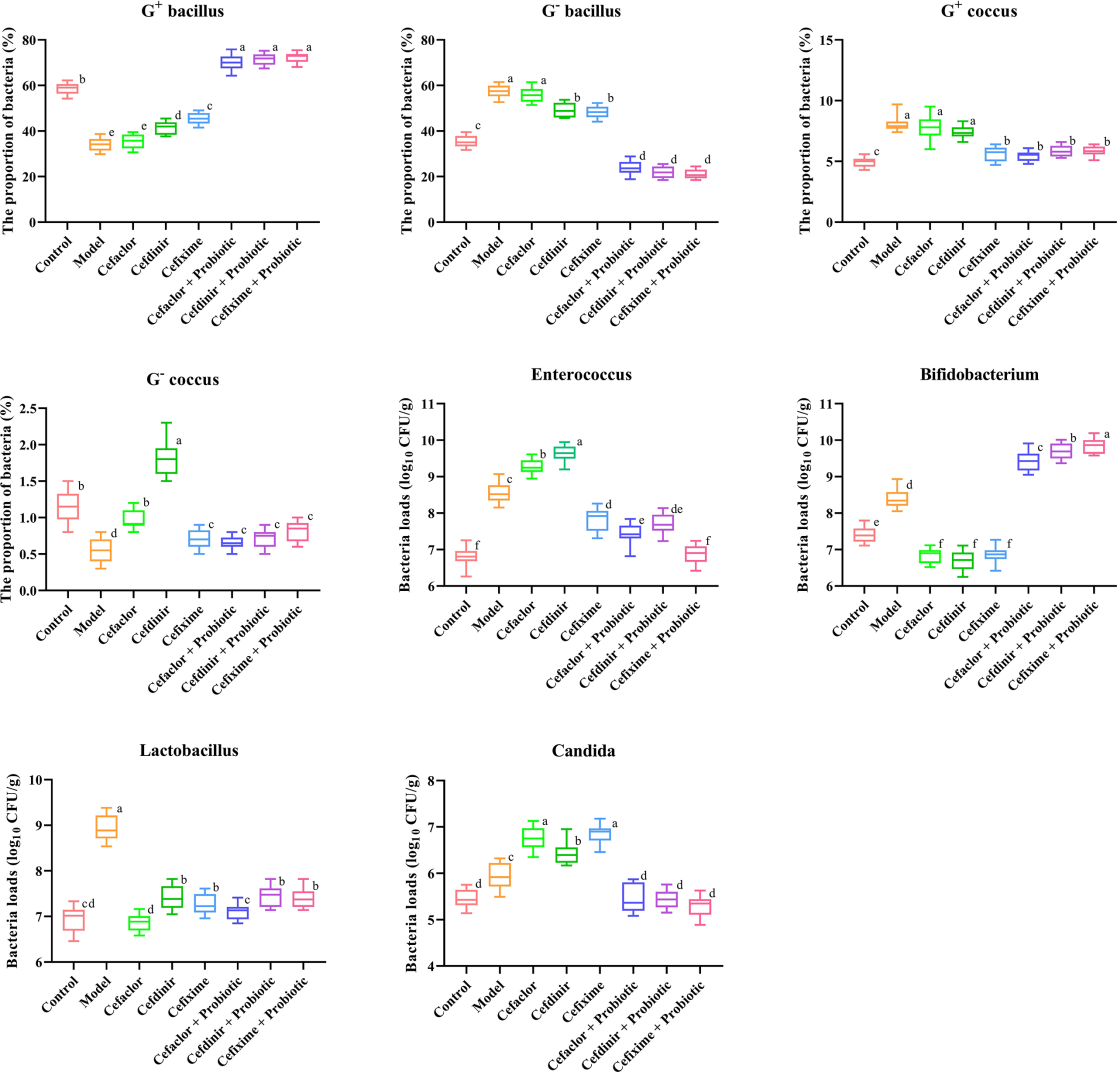
Effects of cephalosporins on the microbial proportions and compositions in the intestinal contents of Spn-infected mice. Data were showed as mean ± SD. Different letters indicated statistically significant differences, P <0.05.

The results in **Figure 5** showed that after Spn infection, the proportions of G^+^ bacilli and G^-^ cocci to total intestinal bacteria was significantly lower than those in the normal control group (*P*<0.05), while the proportions of G^-^ bacilli and G^+^ cocci were significantly increased. However, those abnormal intestinal microbiota alteration could be completely reversed after cephalosporin treatment. Notably, probiotic intervention dramatically elevated the proportions of G^+^ bacilli to total intestinal bacteria, which was even higher than that in the normal control group. In addition, the numbers of *Enterococcus*, *Bifidobacterium*, *Lactobacillus* and *Candida* were significantly higher after Spn infection compared with those in the normal control group (*P*<0.05). The number of *Enterococcus* was significantly increased after 10 days of cefaclor and cefdinir treatment, but decreased after cefixime treatment compared with the model control group (*P*<0.05). Both the number of *Bifidobacterium* and *Lactobacillus* was remarkably elevated after the application of cefixime compared to the normal control group, but the difference was not statistically significant (*P*>0.05); the number of *enterococci* was significantly reduced after the intervention with cephalosporins compared with the model group (*P*<0.05). However, the number of Candida was significantly increased after 10 days of treatment with cefaclor, cefdinir and cefixime compared with the that in the intestinal contents of mice after Spn infection (*P*<0.05). Compared with those in the cephalosporins-treated groups, except the levels of Bifidobacterium were increased, other 3 bacteria were significantly decreased in the intestine of probiotic-treated groups.

To further investigate the changes of intestinal microbiota after antibiotic treatment, the composition of resistant bacteria and opportunistic pathogens was detected by using bacterial auto-analyzer. *Escherichia coli* and *Enterococcus faecalis* were detected in the normal control group, but *Klebsiella pneumoniae*, *Proteus bacilii*, *Pseudomonas aeruginosa* and *Enterococcus faecium* could not be found. After Spn infection, the detection of both *K. pneumoniae* and *P. bacilii* in the model group was observed in 2 out of 10 cases. Therefore, *E. coli* and *E. faecalis* were the dominant bacteria in the intestine of all the groups. Compared with the model group, the number of *E. coli* detected after 10 days of treatment with cefaclor, cefdinir and cefixime were 8, 7 and 8, respectively, and the detection was decreased to 80%, 70% and 80% respectively. The detection rate of *E. faecalis* also showed most significant reduction after cefdinir treatment, which fell by half. More importantly, the detection of *K. pneumoniae*, *P. bacilii*, *P. aeruginosa* and *E. faecium* in cephalosporin-treated groups was sharply increased compared with those in the model group, indicating the exacerbation of intestinal dysbiosis. However, probiotic intervention could obviously reduce the detection of those pathogenic bacteria (**Supplementary Table S2**).

### Cephalosporins promoted pulmonary endothelial barrier disruption in streptococcus pneumoniae-infected mice

**Figure 6:**
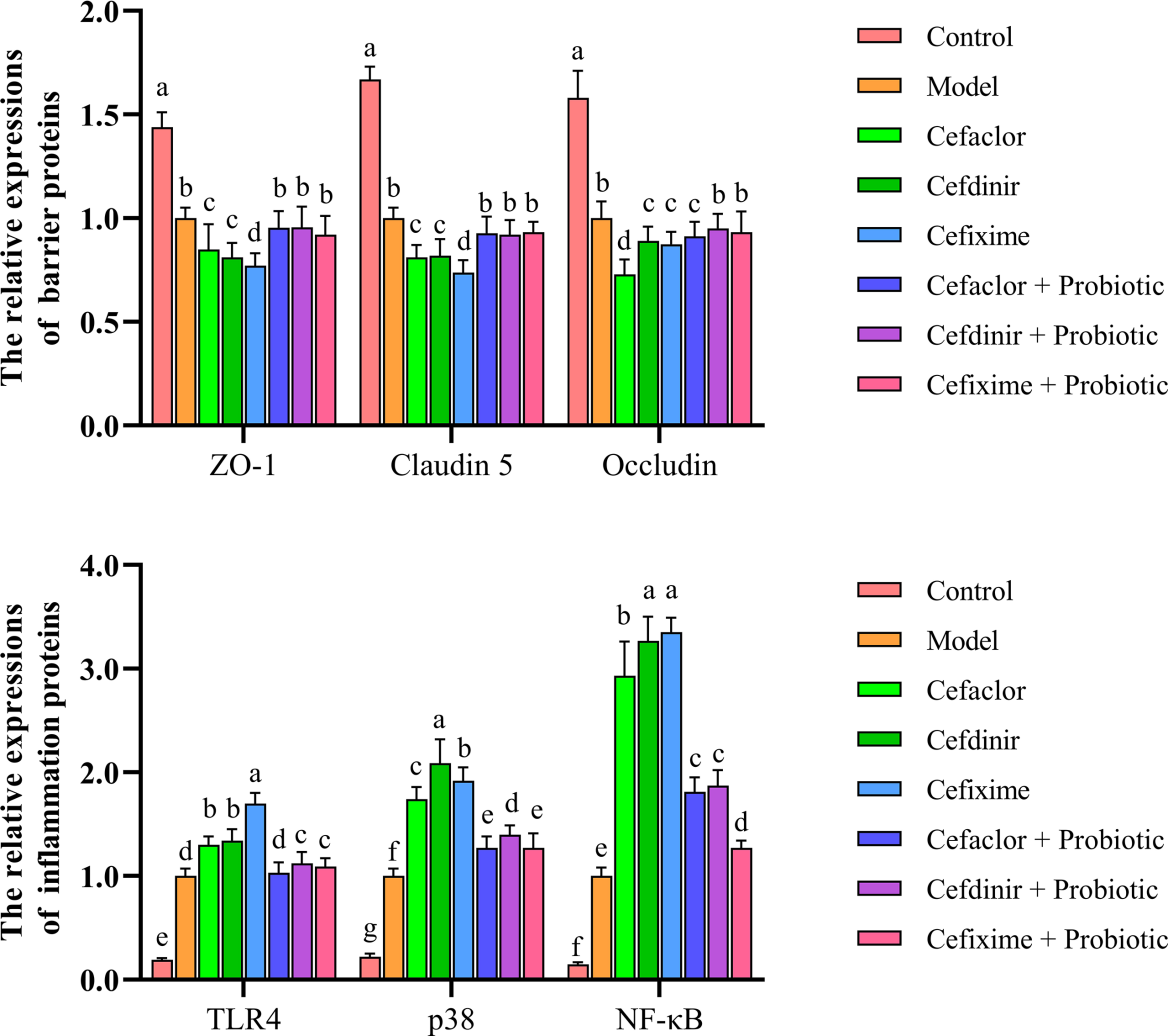
Effects of cephalosporins on expressions of ZO-1, Claudin 5, Occludin, TLR4, p38 and NF-κB mRNAs in the lung tissues of Spn-infected mice. Data were showed as mean ± SD. Different letters indicated statistically significant differences, P <0.05.

**Figure 7:**
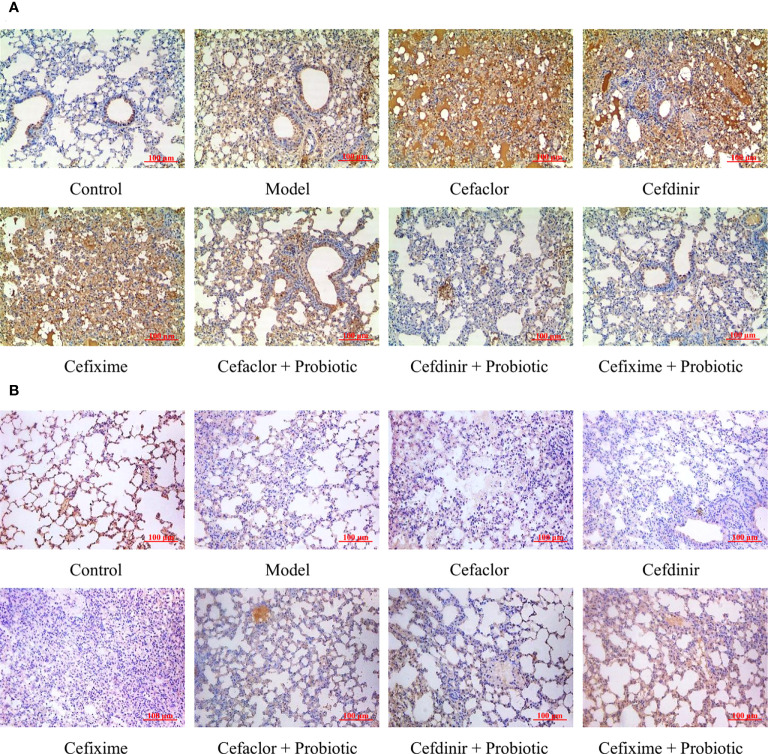
Effects of cephalosporins on **(A)** TLR4 and **(B)** ZO-1 expressions in the lung tissues of Spn-infected mice.

After Spn infection, the expressions of endothelial barrier-related genes (ZO-1, Claudin 5 and Occludin) in the lung tissues of the mice were remarkably reduced whereas the Pathogen-associated molecular pattern (PAMP) genes (TLR4, p38 and NF-κB) were remarkably elevated (*P*<0.05) compared with those in the control mice, indicating the pulmonary endothelial barrier disruption induced by Spn (**Figure 6**). Cephalosporin treatment intensified the trend of those gene expression gaps. But intervention with probiotic could reverse those trends. The immunohistochemical results of TLR4 and ZO-1 protein expressions in the lung tissues of Spn-infected mice were also consistent with the abnormal profiles of those 6 mRNAs (**Figure 7**). Therefore, those results indicated inhibition of probiotic on cephalosporin-deteriorated pulmonary endothelial barrier disruption.

### Cephalosporins altered the intestinal microbial metabolites

**Figure 8:**
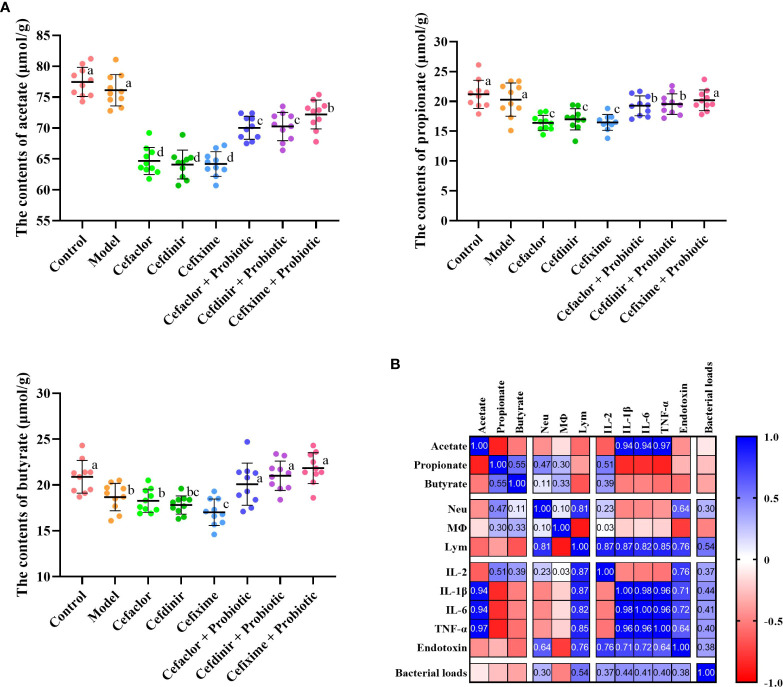
Short-chain fatty acids (SCFA) production and the correlation with disease indicators. **(A)** The concentrations of SCFA in intestinal contents of infected mice. **(B)** The correlation between SCFA and disease indicators. Neu, neutrophils; Mφ, macrophages; Lym, lymphocytes. Data were showed as mean ± SD. Different letters indicated statistically significant differences, P <0.05.

After 7 days of Spn infection, the concentrations of microbial metabolites (acetate, propionate and butyrate) in the intestinal contents of the mice were significantly decreased, while cephalosporin treatment accelerated those declining trends (**Figure 8**). But intervention with probiotic significantly increased the concentrations of acetate, propionate and butyrate in the intestinal contents of the mice. Interestingly, by Pearson correlation analysis, the concentrations of intestinal acetate seemed to be closely related to the serum pro-inflammatory cytokines, indicating that antibiotic and probiotic might impact the lung inflammation in Spn-infected mice partly *via* mediating the production of intestinal acetate.

## Discussion

The development and application of antibiotics has led to a significant reduction in mortality from infection-related diseases, but the widespread use of broad-spectrum antibiotics has also led to increasing bacterial resistance to antibiotics. The problem of bacterial resistance has become an increasingly serious global public health issue (Mc Carlie et al., 2020). The intestinal microbiota is an important component of the intestinal environment, regulating intestinal homeostasis through interactions with the host, and has important immunomodulatory, endocrine and metabolic functions. The disruption of the intestinal micro-ecological barrier under the pressure of broad-spectrum antibiotics may not only allow exogenous conditioned pathogenic bacteria to colonize the intestinal tract, but may also induce intestinal origin of conditioned pathogenic bacteria to cause intestinal-derived infections (Kim et al., 2017; Dhar and Mohanty, 2020; Yang et al., 2021).

Spn is an important pathogen of community-acquired infections in children and a common cause of respiratory infections in children (Leung et al., 2018). Oral cephalosporins are commonly used in the clinical management of Spn infections in children. Data from clinical trials have shown that cephalosporins has a significant impact on the proportion and composition of human intestinal microbiota. In addition, early application of broad-spectrum antibiotics can interfere with the colonization of the normal intestinal microbiota in infants and young children, disrupting the balance of the microbiota established early in life and making it easier for a few conditional pathogenic bacteria to translocate and develop drug resistance, the younger the age, the more pronounced the effects (Zimmermann and Curtis, 2019; Gamage et al., 2021). Also, the use of broad-spectrum antibiotics in infancy leads to an increased incidence of allergic disease, and that the high incidence of allergic disease in children is associated with an imbalance in the intestinal microbiota due to changes in the intestinal microbiota caused by the use of antibiotics (Fernandez et al., 2014; Esposito et al., 2016). Therefore, it is important to investigate the effect of cephalosporins on the proportion and composition of intestinal microbiota during the treatment of common infectious diseases in children, in order to guide the rational use of drugs in clinical practice.

In this study, the traditional isolation and detection method was still adopted, in which the feces and intestinal contents of mice were collected for smear observation and bacterial culture counting. The number of cocci and bacilli in the fecal smear is a basic indicator of the balance of the intestinal microbiota. In the pneumonia model of Spn infection, continuous application of cefaclor, cefdinir or cefixime for 10 days could affect the proportion of cocci and bacilli in the intestinal microbiota. It is well known that the normal intestinal microbiota in the human body includes aerobic, parthenogenic and anaerobic bacteria, of which, anaerobic bacteria are absolutely dominant. *Lactobacillus*, *Bifidobacterium* and other specific anaerobic bacteria in the intestine are planted on the epithelial surface of the intestinal mucosa, and are physiological probiotics of the body (Maldonado Galdeano et al., 2019; Rose et al., 2021). In a variety of disease states or with the use of antibiotics, the intestinal probiotic bacteria are reduced, and the intestinal aerobic and partly aerobic bacteria proliferate and become the dominant microbiota, resulting in the intestinal dysbiosis. Given the fact that the intestinal microbiota is complex and diverse in terms of species and numbers, in this study, common parthenogenic aerobic bacteria, *Enterococci*, *Bifidobacteria*, *Lactobacilli* and fungi were selected as subjects. It was shown that the application of cephalosporins in Spn-induced pneumonia model caused a significant decrease in the relative abundance of *Bifidobacterium* and *Lactobacillus*, but a significant increase in *Enterococci* and *Candida*. This finding was consistent with the previous reports that long-term use of ceftriaxone might lead to the production of drug-resistant and conditionally pathogenic bacteria and replace beneficial bacteria as the dominant microbiota (Harris et al., 2019; Yuan et al., 2019). In the present study, we also observed the changes in intestinal parthenogenic aerobic bacteria, enterococci and fungal species. Our study demonstrated that E. coli and E. faecalis were the dominant bacteria in the intestine, and other conditional pathogens were hardly detected by conventional plate culture methods since their numbers were below the sensitivity of detection. However, after the application of cephalosporins, conditional pathogens such as *K. pneumoniae*, *P. bacilii*, *P. aeruginosa* and *E. faecium* were presented to varying degrees, but *B. bifidum* intervention could partly reverse those alterations. It might be related to a reduction in the number of *Lactobacillus*, resulting in reduced resistance to their colonization. It indicated that the common pathogens of community-acquired infections, such as *K. pneumoniae*, *P. bacilii*, *P. aeruginosa* and *E. faecium*, might in part be of enteric origin, and maintaining intestinal microbiological homeostasis could provide a new strategy to control community-acquired infections in clinic.

The blood-gas barrier is composed of alveolar epithelial cells and pulmonary microvascular endothelial cells. The integrity of the barrier function presents the indispensable ability for maintaining the gas exchange of the lung. During sepsis-induced damage to the blood-gas barrier, there is obvious attenuation and translocation of the tight junction proteins (including ZO-1, Claudin 5 and Occludin) in the alveolar epithelial cell membrane, demonstrating that the tight junctions are disrupted (Shirvaliloo, 2021; Pang et al., 2022). These can cause alveolar oedema, persistent hypoxia and a deteriorating prognosis (Mittal et al., 2014). Therefore, maintaining the integrity of the tight junctions in the alveolar epithelium could be a potential strategy for the treatment of respiratory tract infection (Abedi et al., 2020). Toll-like receptors are considered to be the key interface among the intestinal epithelial barrier, the microbiota and the immune system. TLR4, as the only receptor that recognizes pathogen-associated molecular patterns, acts as a ‘switch’ in this process and is one of the main receptors that activate intrinsic immunity. In normal lung tissue, low TLR4 expression ensures a controlled state of inflammation. In Contrast, in Spn-infected patients, increased expression of TLR4 is sustained, resulting in inappropriate signaling by lung epithelial cells to LPS, through activating various molecules including MyD88, which ultimately activates the NF-κB signaling pathway and boosts the excessive release of TNF-α and other pro-inflammatory cytokines, resulting in the blood-gas barrier damage and lung epithelial cell apoptosis (Aboudounya and Heads, 2021; Tang et al., 2021; Hao et al., 2022). In this study, as the serum levels of pro-inflammatory cytokines and endotoxin were increased, the expressions of ZO-1, Claudin 5 and Occludin in the lung tissues of Spn-infected mice were significantly decreased while TLR4, p38 and NF-κB were increased. Pre-treatment with cephalosporins not only promoted the growth of conditional pathogenic bacteria, but also remarkably disrupted pulmonary endothelial barrier function. However, probiotic treatment protected against cephalosporin-induced lung endothelial barrier dysfunction and intestinal dysbiosis.

In summary, long-term oral administration with cephalosporins could aggravate Spn-induced lung injury in mice *via* disrupting the intestinal microbiological homeostasis and triggering inflammatory processes. The use of antibiotics in clinics should be rational for different indications, and the duration of antibiotic use must be strictly controlled to avoid allergic and immune disorders caused by intestinal dysbiosis. Probiotic supplementation could be considered to reduce the adverse effects of intestinal dysbiosis and prevent bacterial pulmonary inflammation. Therefore, this study also provides new ideas for the prevention and treatment of clinical bacterial infectious diseases.

## Data availability statement

The datasets presented in this study can be found in online repositories. The names of the repository/repositories and accession number(s) can be found below:

https://www.jianguoyun.com/p/DS4TYOgQ4qnjChifiMwEIAA.

## Ethics statement

The animal study was reviewed and approved by Hangzhou Medical College.

## Author contributions

H-ZY conceived and directed the study. C-YS, and J-FW did the analysis and visualization. C-YS, and J-FW drafted the manuscript. H-ZY revised the manuscript. All authors contributed to the article and approved the submitted version.

## Funding

This work is supported by National Natural Science Foundation of China (No. 81673583) and Medical Science of Zhejiang Province (No. 2020KY527).

## Conflict of interest

The authors declare that the research was conducted in the absence of any commercial or financial relationships that could be construed as a potential conflict of interest.

## Publisher’s note

All claims expressed in this article are solely those of the authors and do not necessarily represent those of their affiliated organizations, or those of the publisher, the editors and the reviewers. Any product that may be evaluated in this article, or claim that may be made by its manufacturer, is not guaranteed or endorsed by the publisher.
