# Supplementary material for: Cephalosporins-induced intestinal dysbiosis exacerbated pulmonary endothelial barrier disruption in streptococcus pneumoniae-infected mice
Source: Front Cell Infect Microbiol. 2022 Aug 24;12:997368. doi: 10.3389/fcimb.2022.997368 (PMC9449322; doi:10.3389/fcimb.2022.997368)
Supplement: Supplementary file 1 [file Table_1.docx]

Table 1 The sequences of the primers

| Primers | Sequences (5′→3′) |
| --- | --- |
| Claudin 5 | F: TGGTGCTGTGTCTGGTAGGATGG |
|  | R: GTCACGATGTTGTGGTCCAGGAAG |
| ZO-1 | F: AGCTGCCTCGAACCTCTACTCTAC |
|  | R: GCCTGGTGGTGGAACTTGCTC |
| Occludin | F: TGGCTATGGAGGCGGCTATGG |
|  | R: AAGGAAGCGATGAAGCAGAAGGC |
| TLR4 | F: GGAACAGACCAACTATCGGC |
|  | R: GAGACAACCACTACCATCCG |
| p38 | F: GGAGGTGC-CCGAACGATAC |
|  | R: TTGGCGTGAATGATGGACTG |
| NF-κB | F: CGACTGGTTCACTGCTCCTAATCC |
|  | R: ATGCTGGCTGCTGCTTCACTG |
| GAPDH | F: AAGAAGGTGGTGAAGCAGGCATC |
|  | R: CGGCATCGAAGGTGGAAGAGTG |

Table 2 The detection rate of bacteria in intestinal contents

| Group | n | Escherichia coli | Enterococcus faecalis | Klebsiella pneumoniae | proteus bacilii | Pseudomonas aeruginosa | Enterococcus faecium |
| --- | --- | --- | --- | --- | --- | --- | --- |
| Control | 10 | 10 | 10 | 0 | 0 | 0 | 0 |
| Model | 10 | 10 | 10 | 2 | 2 | 0 | 0 |
| Cefaclor | 10 | 8 | 6 | 2 | 3 | 4 | 2 |
| Cefdinir | 10 | 7 | 5 | 2 | 4 | 3 | 3 |
| Cefixime | 10 | 8 | 8 | 2 | 2 | 3 | 2 |
| Cefaclor + Probiotic | 10 | 9 | 8 | 1 | 0 | 1 | 0 |
| Cefdinir + Probiotic | 10 | 9 | 7 | 0 | 1 | 1 | 1 |
| Cefixime + Probiotic | 10 | 10 | 9 | 0 | 0 | 1 | 0 |
